# Safeguarding future generations: a One Health perspective on children, climate change, and infectious threats

**DOI:** 10.3389/fpubh.2026.1771844

**Published:** 2026-03-06

**Authors:** Marco Masetti, Francesca Lato, Martina Menoni, Susanna Esposito

**Affiliations:** Department of Medicine and Surgery, Pediatric Clinic, University Hospital, University of Parma, Parma, Italy

**Keywords:** antimicrobial resistance, child health, climate change, emerging infectious diseases, One Health, vector-borne diseases

## Abstract

The One Health approach recognizes the interconnectedness of human, animal, and environmental health, offering a critical framework for addressing complex global health challenges. Children occupy a uniquely vulnerable position within this paradigm due to their physiological immaturity, developmental sensitivity, behavioral exposures, and dependence on surrounding ecosystems. This narrative review examines how major contemporary threats—antimicrobial resistance (AMR), climate change, and emerging infectious diseases—intersect to shape child health outcomes within a One Health perspective. We synthesize evidence from human, animal, and environmental health domains to illustrate how children are disproportionately exposed to resistant pathogens, climate-sensitive hazards, and zoonotic and vector-borne infections. Particular attention is given to pediatric and neonatal AMR, climate-related impacts on physical and mental health, and the expanding geographic range of vector-borne diseases affecting children. The review highlights how factors such as antibiotic use in humans and animals, environmental contamination, urbanization, biodiversity loss, and extreme weather events converge to amplify risks during critical developmental windows. We identify major gaps in child-specific surveillance, integrated research, and policy implementation, especially in low- and middle-income countries. We argue that embedding a child-centered lens within One Health research, governance, and interventions is essential to protect current and future generations. Advancing such an integrated approach can enhance prevention, strengthen health system resilience, and promote equity in an era of escalating ecological and infectious threats.

## 1 Background

In recent decades, the concept of One Health—an integrative approach recognizing the interdependence of human, animal, and environmental health—has gained increasing prominence as a framework for addressing complex and interrelated global health challenges (1). Initially developed to better understand and prevent zoonotic disease emergence at the human–animal interface, the One Health paradigm has progressively expanded to encompass broader environmental, social, and ecological determinants of health. Within this evolving framework, there is growing recognition that children occupy a uniquely vulnerable and strategically important position (2).

Children represent one of the populations most susceptible to health risks arising from environmental degradation, ecosystem disruption, food-system instability, and infectious disease spillover. Their physiological, developmental, and behavioral characteristics—including higher intake of air, water, and food per unit body weight, immature immune and detoxification systems, and ongoing organ development—render them particularly sensitive to cumulative exposures and multifactorial health threats (2, 3). Conditions such as childhood stunting, which continues to affect nearly one quarter of children under 5 years of age worldwide, exemplify the need for a One Health–oriented response, as their underlying drivers span human nutrition, animal agriculture, environmental contamination, food security, and ecosystem health (4, 5).

Despite this clear rationale, child health programs have historically been organized within discipline-based silos, separating human health from veterinary and environmental health. This fragmentation has limited the capacity to address the interconnected nature of children's risk exposures. As recently emphasized, meaningful improvements in the lives of millions of children living in poverty require approaches that explicitly recognize these interdependencies and apply One Health methods in practice (5).

There is therefore an urgent need to embed a One Health lens within child health research, policy, and clinical practice. Such an approach offers opportunities not only to strengthen protection against zoonoses and environmental hazards, but also to leverage synergies in prevention, early intervention, and health promotion across the life course, from the prenatal period through adolescence. Moreover, engaging children and adolescents as active stakeholders in One Health—through education, behavioral literacy, and community-based environmental health initiatives—may generate long-term benefits for resilience, ecosystem stewardship, and sustainable development (6, 7). Importantly, the burden of environmental and zoonotic risk is borne disproportionately by children in low- and middle-income countries and socially disadvantaged settings; any child-focused One Health agenda must therefore prioritize equity, context-specific solutions, and cross-sectoral justice (2, 4, 8).

In this narrative review, we examine the intersections between children's health and the One Health approach, with particular attention to climate change, emerging infectious diseases, and antimicrobial resistance. We explore how the human–animal–environment interface shapes pediatric health risks, summarize the available evidence on integrated interventions, and identify critical research and operational gaps. We argue that advancing a child-centered One Health agenda is both timely and essential to safeguarding the health of future generations in an era of escalating ecological and zoonotic threats.

## 2 Methods

This narrative review was conducted to synthesize current evidence on emerging and evolving health risks affecting children—specifically antimicrobial resistance (AMR), climate change, and emerging infectious diseases—within a One Health framework. The objective was to explore how interactions among human, animal, and environmental health influence pediatric vulnerability and disease patterns.

An electronic literature search was performed using the PubMed database, including publications available up to August 2025. The search strategy combined terms related to vectors (e.g., “vector,” “mosquito”), pathogens (e.g., “virus,” “bacteria,” “parasite”), specific diseases (e.g., “West Nile,” “dengue,” “Zika,” “chikungunya”), and climate- and environment-related factors (e.g., “climate change,” “urbanization,” “deforestation,” “natural disasters,” “global warming,” “temperature,” “precipitation”). These terms were paired with epidemiological, clinical, and population-specific keywords such as “infectious disease,” “antimicrobial resistance,” “health,” “policy,” “pediatric,” “child,” and “neonate.” Boolean operators (AND, OR) were used to optimize search sensitivity and specificity.

Only peer-reviewed articles published in English were included. Eligible sources comprised original research articles, systematic reviews, meta-analyses, surveillance reports, and relevant clinical and epidemiological studies. Non–peer-reviewed literature, editorials, and commentaries were excluded. Reference lists of selected articles and key reviews were manually screened to identify additional relevant publications not captured by the initial search.

Although the search strategy included multiple vector types, the review was deliberately restricted to arboviral infections and selected parasitic diseases (e.g., malaria and leishmaniasis) to maintain thematic focus. Other emerging infections, such as mpox, were not included due to their distinct epidemiological characteristics and the need for more extensive, dedicated analysis.

Titles and abstracts were independently screened for relevance by two reviewers. Full texts of potentially eligible articles were subsequently assessed for inclusion. Discrepancies were resolved through discussion or consultation with a third reviewer. From each included study, data were extracted on key variables of interest, including population characteristics, exposure pathways, epidemiology, interventions, and reported outcomes.

The selected evidence was analyzed qualitatively and organized thematically by topic area, intervention type, epidemiological patterns, and health outcomes. This approach enabled an integrated synthesis of findings across disciplines, including pediatric infectious diseases, epidemiology, climatology, and environmental health, consistent with a One Health perspective.

## 3 Results

### 3.1 Antimicrobial resistance and children

AMR is widely recognized as one of the most pressing global health threats, undermining the effectiveness of antibiotics across human, animal, and environmental domains (9, 10). The World Health Organization (WHO) has identified AMR as one of the top 10 global public health threats facing humanity (11). While much of the scientific, clinical, and policy discourse has traditionally focused on adult populations and hospital-acquired infections, children represent a uniquely vulnerable and often under-recognized population within the AMR landscape.

Children's developing physiology, frequent exposure to infectious diseases, high rates of antibiotic prescribing, and close interactions with animals and the environment place them squarely at the intersection of human, animal, and environmental health—the core triad of the One Health paradigm (Table 1). Their immature immune systems, age-specific behaviors, and cumulative exposures increase susceptibility not only to infection but also to colonization and disease caused by resistant organisms (2, 3). Embedding pediatric AMR within a One Health framework therefore provides critical insight into the multidimensional drivers of resistance and offers opportunities to design more effective, integrated prevention and control strategies.

**Table 1:** Pediatric antimicrobial resistance: One Health drivers, exposure pathways, and policy needs.

**Theme**	**Key points**
Child vulnerability	Developing immune system, frequent infections, high antibiotic exposure.
Antimicrobial resistance burden	Rising resistance in key pathogens (Enterobacteriaces, ESBL, carbapenem-resistant strains).
Exposure pathways	Antibiotic use, environmental reservoirs, animal contact, food chain.
One Health relevance	Interactions across humans, animals, and environment drive resistance.
Drivers of resistance	Empiric prescribing, animal antibiotic use, environmental contamination, socio-behavioral factors.
Policy implications	Need for child-specific surveillance, stewardship, environmental hygiene, and cross-sector governance.

Globally, children experience a high burden of infectious diseases, and antibiotic use in pediatric populations is correspondingly common, often empiric and not always aligned with microbiological evidence. Although pediatric-specific AMR surveillance remains limited in many regions, emerging data indicate rising resistance rates in key pathogens affecting children. For example, analysis from the China Antimicrobial Surveillance Network (CHINET) reported increasing prevalence of extended-spectrum β-lactamase (ESBL)–producing and carbapenem-resistant *Enterobacterales* among pediatric isolates between 2015 and 2021 (12). Importantly, children's exposure to resistant bacteria is not limited to clinical antibiotic use; environmental reservoirs and zoonotic or food-chain pathways also play a significant role. This convergence of clinical, environmental, and animal-related exposures in early life underscores the necessity of a holistic One Health perspective (13).

Despite the high burden of AMR and infectious diseases in low- and middle-income countries (LMICs), implementation of pediatric antimicrobial stewardship programs (ASPs) in these settings remains limited and uneven. Structural constraints such as shortages of trained personnel, limited diagnostic capacity, high patient volumes, and fragmented healthcare delivery pose significant challenges (14). However, stewardship interventions can be adapted to LMIC contexts through pragmatic, stepwise approaches that build on existing child health platforms rather than relying on resource-intensive, hospital-based models alone (15). Integration of ASP principles into established programs, including Integrated Management of Childhood Illness (IMCI), neonatal sepsis pathways, immunization services, and nutrition programs, may represent a feasible and scalable strategy (16). Embedding standardized treatment guidelines, age- and weight-appropriate dosing tools, and clear referral criteria into routine pediatric care can support more rational antibiotic use even in settings with limited specialist availability.

Community-based approaches are particularly relevant for pediatric stewardship in LMICs, where a substantial proportion of antibiotic exposure occurs outside hospital settings (17–19). Community health workers can play a central role in caregiver education, early identification of severe illness, reinforcement of adherence to prescribed regimens, and discouragement of inappropriate antibiotic use for self-limiting infections. Their involvement may help address key behavioral and access-related drivers of antibiotic misuse at the household and community level. Incorporating stewardship messaging into routine community outreach activities, maternal and child health visits, and health education campaigns may therefore enhance acceptability, equity, and sustainability of pediatric ASPs in resource-constrained settings (19).

Although published evidence on pediatric ASPs in LMICs remains limited, available reports suggest that stewardship interventions are both feasible and beneficial. Hospital-based initiatives in neonatal and pediatric units in sub-Saharan Africa and South Asia, often centered on locally adapted treatment guidelines, prospective audit and feedback, and restriction of selected broad-spectrum antibiotics, have demonstrated reductions in antibiotic initiation, duration of therapy, and use of last-line agents without increases in mortality or adverse outcomes. Importantly, several of these interventions were implemented with minimal additional resources and relied on multidisciplinary collaboration, highlighting that effective stewardship does not necessarily require high-cost infrastructure (15, 19, 20). Nevertheless, most published studies are single-center or short-term, underscoring the need for robust evaluation, longer follow-up, and multicenter implementation research focusing on pediatric-specific outcomes.

Strengthening laboratory infrastructure, surveillance systems, and cross-sector collaboration is essential to support sustainable pediatric ASP in LMICs. Access to basic microbiological diagnostics, timely reporting of culture and susceptibility results, and age-disaggregated AMR surveillance data are critical for informing empiric therapy and monitoring the impact of stewardship interventions (21). Where comprehensive laboratory services are not immediately feasible, phased approaches such as sentinel surveillance, simplified diagnostic algorithms, and regional laboratory networks may provide actionable data. Integration of human health surveillance with animal and environmental AMR monitoring, consistent with a One Health framework, can further improve understanding of resistance transmission pathways relevant to children (21). Coordinated investment, policy support, and collaboration across health, veterinary, and environmental sectors will be necessary to ensure that pediatric ASPs contribute meaningfully to reducing AMR burden and improving child health outcomes in LMICs.

#### 3.1.1 Transmission pathways at the human–animal–environment interface

A central tenet of the One Health paradigm is that antimicrobial-resistant bacteria and antimicrobial resistance genes (ARGs) circulate dynamically among humans, animals, and the environment. Evidence from animal husbandry, wildlife, and companion-animal settings demonstrates that resistant organisms selected in animals can enter human populations through direct contact, contaminated food products, or environmental pathways such as manure, soil, and water (22). A One Health–oriented review of zoonotic and antibiotic-resistant Campylobacter illustrated shared resistance profiles and genetic determinants across animal, food, and human isolates, highlighting the interconnected nature of transmission across sectors (23).

In children, these pathways may be amplified. Children frequently play outdoors, engage in hand-to-mouth behaviors, interact with domestic animals, and in many settings live in close proximity to livestock or consume animal-derived foods that may harbor resistant organisms or antibiotic residues. A longitudinal cohort study protocol from rural India explicitly adopted a One Health design to examine *Escherichia coli* resistance patterns in children alongside household drinking water, domestic animals, and environmental water sources, reflecting the complexity of interconnected exposure pathways in pediatric populations (20).

The drivers of AMR in children overlap with, but also extend beyond, those observed in adults. First, high rates of empiric antibiotic prescribing in pediatric outpatient and inpatient settings—often in the absence of rapid diagnostics—generate strong selective pressure for resistance (24). Second, antimicrobial use in food-producing animals for growth promotion, prophylaxis, or therapy remains a major driver of resistance emergence, facilitating transfer of resistant bacteria and genes to humans via food chains and environmental contamination (25). Third, environmental dissemination of antibiotics, metabolites, resistant bacteria, and ARGs through wastewater, agricultural runoff, and soil contributes to a growing environmental “resistome” to which children are disproportionately exposed, particularly in settings with inadequate sanitation (26).

Socio-behavioral factors further shape children's AMR risk. Caregiver health-seeking behaviors, informal access to antibiotics, animal husbandry practices, and household hygiene conditions all influence exposure and transmission dynamics. The Indian cohort study cited above aimed to link caregiver behaviors, prescribing patterns across formal and informal providers, and resistance profiles in children, animals, and water sources, underscoring the relevance of integrated, community-level approaches (17). These risks are exacerbated in low- and middle-income countries (LMICs), where gaps in health systems, diagnostics, antibiotic stewardship, sanitation infrastructure, and regulation of animal production limit effective mitigation (9, 26).

#### 3.1.2 Antimicrobial resistance in neonates and children within a One Health perspective

Although pediatric AMR research explicitly framed within a comprehensive One Health framework remains limited, a growing body of evidence from neonatal and early childhood studies highlights the severity of the problem and the urgent need for integrated, cross-sectoral interventions (17, 27, 28). Neonates are particularly vulnerable, as their immature immune systems, frequent exposure to invasive procedures, and prolonged hospital stays increase the risk of colonization and infection with multidrug-resistant organisms (MDROs) (29, 30). Neonatal intensive care units (NICUs) can be viewed as microcosms of the One Health system, where clinical practices, environmental hygiene, and microbial ecology intersect (31, 32) (Table 2).

**Table 2:** Antimicrobial resistance in neonates and children within a One Health perspective.

**Areas**	**Evidence**	**Implications/actions**
Neonatal vulnerability	Neonates' immature immune systems, invasive procedures, and prolonged hospitalization increase susceptibility to multidrug-resistant organism (MDRO) infection and colonization.	Strengthen infection-prevention and control (IPC) practices, ensure functional hygiene infrastructure, and improve protective equipment and staffing in neonatal units.
Clinical–environmental interface	Neonatal intensive care units (NICUs) act as microcosms of the One Health system, linking patient care, microbial ecology, and environmental contamination.	Integrate environmental surveillance into NICU routines and implement routine environmental cleaning and monitoring of surfaces and sinks.
Global antimicrobial resistance burden in neonates	High prevalence of multidrug-resistant Gram-negative organisms across regions, particularly in low- and middle-income countries (LMICs).	Develop and enforce local antimicrobial stewardship protocols; improve diagnostic and laboratory capacity to guide targeted therapy.
Colonization reservoirs	Asymptomatic colonization by resistant organisms is common in preterm and hospitalized infants, facilitating ongoing transmission.	Conduct periodic colonization screening and limit cross-transmission through improved hand hygiene and equipment sterilization.
Stewardship gaps	Existing neonatal antimicrobial stewardship programmes (ASPs) reduce antibiotic exposure but have limited demonstrated impact on resistance rates.	Implement One Health–aligned ASPs that integrate clinical, microbiological, and environmental data for more effective resistance control.
Global policy and surveillance	Neonatal AMR remains underrepresented in global AMR monitoring systems and databases.	Expand inclusion of neonatal and pediatric data in global surveillance systems such as GLASS; align with the Tripartite AMR Action Plan.
Research and development priorities	There is a critical shortage of pediatric-specific pharmacokinetic data and a global gap in antibiotic R&D for neonates.	Prioritize investment in pediatric antibiotic research, development, and optimized dosing studies.
One Health integration	The neonatal AMR crisis reflects systemic weaknesses across clinical practice, governance, and environmental hygiene.	Adopt integrated One Health policies that link human, animal, and environmental health; strengthen cross-sectoral collaboration and surveillance.

This complexity is well-illustrated by a mixed-methods study conducted in the NICU of Felege Hiwot Hospital in Ethiopia. Among 420 neonatal and environmental samples collected between October 2022 and June 2023, approximately 35% yielded resistant pathogens (33). The predominant isolates—coagulase-negative *Staphylococci, Klebsiella pneumoniae*, and *Acinetobacter* spp.—showed alarmingly high resistance to gentamicin, cotrimoxazole, and ciprofloxacin (98%−100%), while resistance to amikacin remained relatively low (34). Crucially, the study identified environmental and procedural risk factors, including non-functional sinks, inadequate personal protective equipment, and overcrowding, directly linking resistance patterns to deficiencies in environmental hygiene and infection prevention and control (IPC) (33, 34).

Similar concerns have emerged from studies of neonatal urinary tract infections. A recent retrospective cohort study (2018–2024) reported widespread multidrug resistance among neonatal isolates, with high rates of ampicillin and gentamicin resistance across *Escherichia coli, Klebsiella pneumoniae*, and *Enterobacter* spp. Multidrug resistance affected over half of *E. coli* isolates and all *Enterobacter* isolates, underscoring the urgency of optimizing antimicrobial stewardship and treatment protocols in neonatal care (35).

The burden of neonatal AMR is particularly severe in LMICs. A retrospective study from a tertiary NICU in Pune, India, documented high resistance rates among Gram-negative pathogens, including resistance to aminoglycosides (74%), third- and fourth-generation cephalosporins (95%), and carbapenems (56%), with colistin resistance detected in nearly one-third of K. pneumoniae isolates (36). Low birth weight and invasive device use were associated with increased mortality, highlighting the interaction between host vulnerability, clinical practice, and microbial adaptation (36).

Beyond overt infection, colonization studies reveal substantial subclinical reservoirs of resistance. Screening of very preterm infants (≤32 weeks' gestation) demonstrated colonization rates of up to 38% with resistant Gram-negative organisms, including ESBL- and AmpC-producing strains, emphasizing colonization as a critical but often overlooked component of AMR transmission in NICUs (37).

ASPs tailored to neonates and children are therefore essential. In NICUs, interventions such as prospective audit and feedback, guideline implementation, and restriction of broad-spectrum antibiotics have consistently reduced antibiotic initiation and treatment duration without increasing mortality or relapse (27, 38, 39). A meta-analysis encompassing more than 350,000 neonates reported a 19% reduction in antibiotic starts and a nearly 2-day reduction in treatment duration following ASP implementation (39). Long-term single-center data further demonstrate sustained reductions in antibiotic days of therapy, particularly for commonly used agents (40). Nevertheless, systematic reviews highlight persistent gaps, including limited evaluation of ASP effects on resistance rates and healthcare-associated infections, as well as insufficient long-term surveillance (38, 41).

Recognizing these challenges, the WHO Pediatric Drug Optimisation (PADO) initiative has identified neonates and children as priority populations for antibiotic research and development, citing critical shortages in pediatric pharmacokinetic data and mismatches between global antibiotic supply and child health needs (42). This framing situates pediatric AMR not only as a clinical concern, but also as a developmental and equity issue spanning multiple sectors.

At the global policy level, progress has been made, but significant gaps remain. The Global Antimicrobial Resistance and Use Surveillance System (GLASS) has begun incorporating pediatric and neonatal data in some countries, although systematic inclusion is inconsistent (43). Initiatives such as the Global Antibiotic Research and Development Partnership (GARDP) prioritize neonatal sepsis and pediatric dosing optimization, fostering collaboration among clinicians, policymakers, and industry (44). Similarly, the Tripartite AMR Action Plan led by WHO, FAO, and WOAH emphasizes integrated surveillance across human, animal, and environmental sectors, a strategy increasingly recognized as essential for protecting children (45, 46).

Within this broader One Health framework, neonatal AMR serves as a sentinel indicator of systemic weaknesses in infection control, antimicrobial governance, and environmental hygiene. Integrating clinical, environmental, and policy perspectives enables a more comprehensive understanding of AMR emergence and transmission pathways affecting children (17, 33).

#### 3.1.3 Implications for child health and One Health policy

The convergence of AMR and One Health in pediatric populations carries important implications for policy and practice. There is an urgent need for pediatric-specific, age-disaggregated surveillance systems that link antibiotic use, prescribing practices, caregiving behaviors, animal contact, environmental exposures, and resistance outcomes. Without such data, children remain largely invisible in AMR monitoring frameworks.

Antimicrobial stewardship programs must be adapted to pediatric populations, accounting for age-specific pharmacokinetics, infection spectra, dosing requirements, and adverse-effect profiles, while also extending beyond the clinical setting to address animal and environmental reservoirs. Effective One Health interventions should include reducing non-therapeutic antibiotic use in animals, promoting safe animal husbandry, monitoring antibiotic residues in food and water, improving sanitation infrastructure, and limiting environmental dissemination of resistant bacteria (22, 23). Public health education targeting caregivers and communities is equally critical, emphasizing responsible antibiotic use, hygiene, safe food and water practices, and awareness of AMR risks.

Ultimately, effective responses require coordinated One Health governance, bringing together human health, veterinary, agricultural, and environmental sectors. Harmonizing infection prevention and control, antimicrobial stewardship, and One Health frameworks has the potential to improve patient safety, slow resistance emergence, and strengthen public health outcomes for children (24).

### 3.2 Climate change and impact on human health

Climate change refers to sustained, long-term alterations in regional and global climate patterns, including shifts in temperature, precipitation, and the frequency and intensity of extreme weather events (47). Although climate variability is influenced by natural drivers (e.g., solar cycles, oceanic circulation, volcanic activity), the dominant contributor to contemporary climate change is human activity, particularly the combustion of fossil fuels, industrial processes, and intensive livestock production (48, 49). These activities increase atmospheric concentrations of greenhouse gases—including carbon dioxide, methane, nitrous oxide, and halogenated gases—and are often accompanied by elevated particulate matter. Together, these emissions enhance the greenhouse effect by trapping heat, driving global warming and downstream climate disruptions (48, 49).

Global warming alters the hydrological cycle and contributes to more frequent and severe heatwaves, droughts, floods, storms, wildfires, and sandstorms. This cascading set of changes is typically encompassed within the broader term “climate change” (48, 49). A clear indicator of anthropogenic influence is the accelerated rise in global temperatures since the Industrial Revolution (49). In the WHO European Region, mean temperature increased by approximately 0.5 °C per decade between 1991 and 2021, exceeding the global average and highlighting the region's heightened warming trajectory (50).

Climate-related exposures affect health across the life course, beginning *in utero*, with both direct (e.g., heat stress, air pollution, extreme events) and indirect pathways (e.g., food insecurity, infectious disease ecology, displacement, and psychosocial stress) (51). Associations have been reported between climate change and cardiovascular, respiratory, metabolic, dermatological, allergic, oncological, and mental health outcomes, with disproportionate impacts among vulnerable groups such as pregnant women, children, older adults, and people with chronic illness (49). Within a One Health perspective, these effects are best understood as emergent consequences of interconnected environmental, animal, and human systems, supporting integrated approaches to risk prevention and adaptation (50, 51).

#### 3.2.1 Rising temperatures

Rising ambient temperatures are among the most visible manifestations of climate change. During May–July 2018, 22% of regions north of 30° latitude experienced temperatures above the 90th percentile, and projections indicate continued warming without effective mitigation (52). Urban populations are particularly exposed due to the “urban heat island” effect, driven by built environments, limited green space, and heat generated by transport and industry (49, 52). Extreme heat is also linked to worsened air quality, although the mechanisms and directionality of this relationship can vary across settings (53).

Heat exposure triggers thermoregulatory responses such as peripheral vasodilation and sweating. Vasodilation redistributes circulating volume, reduces preload, and increases heart rate and cardiac oxygen demand, raising the risk of ischemia and oxidative stress—particularly in individuals with chronic disease. Heat-related dehydration and electrolyte imbalance can precipitate renal injury, while medications that impair thermoregulation may further amplify risk in vulnerable groups (52). Limited autonomy, disability, or psychiatric illness may reduce the ability to adopt protective behaviors and increase heat-related morbidity (52).

Children are especially susceptible because of immature thermoregulation, dependence on caregivers, and a higher surface-area-to-mass ratio that can accelerate heat gain and dehydration (52) (Table 3). Temperature also influences pediatric respiratory health: extreme heat and rapid temperature variation may alter epithelial permeability and mucociliary clearance, promote airway inflammation, and trigger bronchoconstriction via vagal pathways (54). Evidence in pediatrics suggests that daily and inter-daily temperature variability can favor respiratory infections, and humidity has been positively associated with respiratory syncytial virus (RSV) transmission dynamics (55). More broadly, climate-driven shifts in pathogen survival and distribution, combined with heightened pediatric susceptibility, may increase respiratory infection risk in children (56, 57).

**Table 3:** Physiological and environmental health impacts associated with rising ambient temperatures.

**Effect**	**Mechanism**	**Health outcome**
Vasodilation	Heat-induced expansion of blood vessels	Cardiovascular events, renal dysfunction
Dehydration	Fluid loss due to elevated temperatures	Electrolyte imbalances (dyselectrolytemia)
Respiratory epithelium permeability	Altered function of the epithelial barrier	Bronchodilation, increased risk of respiratory infections
Skin hydration and sweat composition	Disruption of thermogenesis and lipogenesis	Dysbiosis, dermatoses
Hormonal imbalance	Increased time indoors, fatigue	Reduced physical activity, obesity, oncological risk
Altered flowering pattern	Shift in plant blooming cycles	Changed allergy seasonality, spread of toxic algae

Temperature and humidity changes also affect dermatological and allergic disease. Variations in skin hydration and sweat composition can disrupt cutaneous homeostasis, contributing to dysbiosis and inflammatory dermatoses such as eczema, psoriasis, acne, and seborrheic dermatitis (48). Mechanistically, sweating and keratinocyte swelling may promote follicular occlusion and create microenvironments that alter microbiota composition; studies have linked specific microbial patterns to common dermatoses (e.g., *Staphylococcus aureus* in atopic dermatitis) (58). Heat exposure may also influence metabolic health by affecting hormonal regulation, thermogenesis, and lipogenesis, while behavioral adaptations (e.g., remaining indoors during heatwaves) can reduce physical activity and contribute to obesity—an established risk factor for several cancers (59, 60).

Indirect effects of warming are likewise relevant. Shifts in flora distribution, longer pollen seasons, and increased indoor mold growth can alter the timing and geography of allergic diseases, including asthma and rhinitis (48, 49, 61, 62). Associations between higher temperatures and allergic manifestations have been described (55). In aquatic ecosystems, warming can facilitate the spread of algal and plankton species into new regions, including toxin-producing organisms with potential human health implications (48).

Table 3 summarizes physiological and environmental health impacts associated with rising ambient temperatures.

#### 3.2.2 Environmental pollution

Climate change and environmental pollution interact bidirectionally: greenhouse-gas–driven warming increases the likelihood of extreme events such as fires and storms, which in turn release additional pollutants and can disperse molds and spores, further degrading air quality (49, 57). Air, water, and soil pollution affect multiple organ systems. The respiratory tract and skin are among the earliest and most consistently exposed interfaces, but cardiovascular, endocrine, metabolic, and oncologic risks are also influenced by environmental contaminants (52, 59–61, 63).

#### 3.2.3 Atmospheric pollution

Atmospheric pollution largely reflects emissions of particulate matter and gases from energy production and consumption, transportation, industrial extraction and manufacturing, and agricultural activities. In 2019, these sectors were key contributors to PM2.5 and PM10 emissions, with agriculture representing a notable source of PM2.5 (64). Air quality assessments commonly focus on six pollutants: particulate matter, carbon monoxide, ground-level ozone, lead, nitrogen dioxide, and sulfur dioxide (56).

Between 2005 and 2019, pollutant emissions declined in many European Union countries due to regulatory measures, technological advances, and improved energy efficiency; however, in 2019 more than 90% of urban EU residents were still exposed to harmful levels of air pollutants (64). Exposure occurs through both outdoor and indoor environments. Indoor pollution includes tobacco smoke (firsthand and secondhand), combustion products from cooking and heating, volatile compounds from household products, and infiltration of outdoor pollutants (48, 54, 61). Allergens, dust, particulates, and gases can impair epithelial barrier function in airways and skin, induce epigenetic changes (including prenatally), and contribute to chronic inflammatory conditions such as asthma, allergic rhinitis, and atopic dermatitis (48, 54, 61). Inhalation of PM10, NO_2_, and ozone has been associated with reduced respiratory function in adults and school-aged children (56, 57, 62, 65). PM2.5 is of particular concern in young children because of its ability to reach distal airways (9).

In pediatric asthma, early-life exposure to tobacco smoke may impair mucociliary clearance, promote airway inflammation, and alter respiratory microbiota, increasing the risk of asthma onset, poor control, and exacerbations. Associations between air pollution and asthma exacerbations have also been reported (61). Beyond asthma, pollution interacts with allergic disease more broadly: children raised in greener, rural environments may develop greater tolerance to environmental exposures, whereas urban polluted settings may favor T-helper 2–skewed immune responses (48). Pollutants can amplify allergic responses by increasing epithelial permeability and activating pro-inflammatory epigenetic pathways, while also modifying allergen structure and potentially enhancing allergenicity (55, 57, 61, 63, 65). Observational work has reported higher allergic symptom burden in urban areas and higher risk of allergic rhinitis among urban residents compared with rural populations (3, 10, 22). Rising CO_2_ may further influence allergy patterns by accelerating plant growth and extending pollen seasons (55, 57, 61, 62, 65). Poor outdoor air quality may also increase time spent indoors, increasing exposure to indoor allergens (e.g., dust mites) and molds, which proliferate under warm, humid conditions. Mold proteases can damage epithelial barriers, and some *Aspergillus* and *Penicillium* species have been linked to severe asthma exacerbations (10, 22). Indoor allergens may elicit stronger allergic responses than seasonal allergens, with dust mites a frequent trigger of allergic asthma (22).

**Figure 1:**
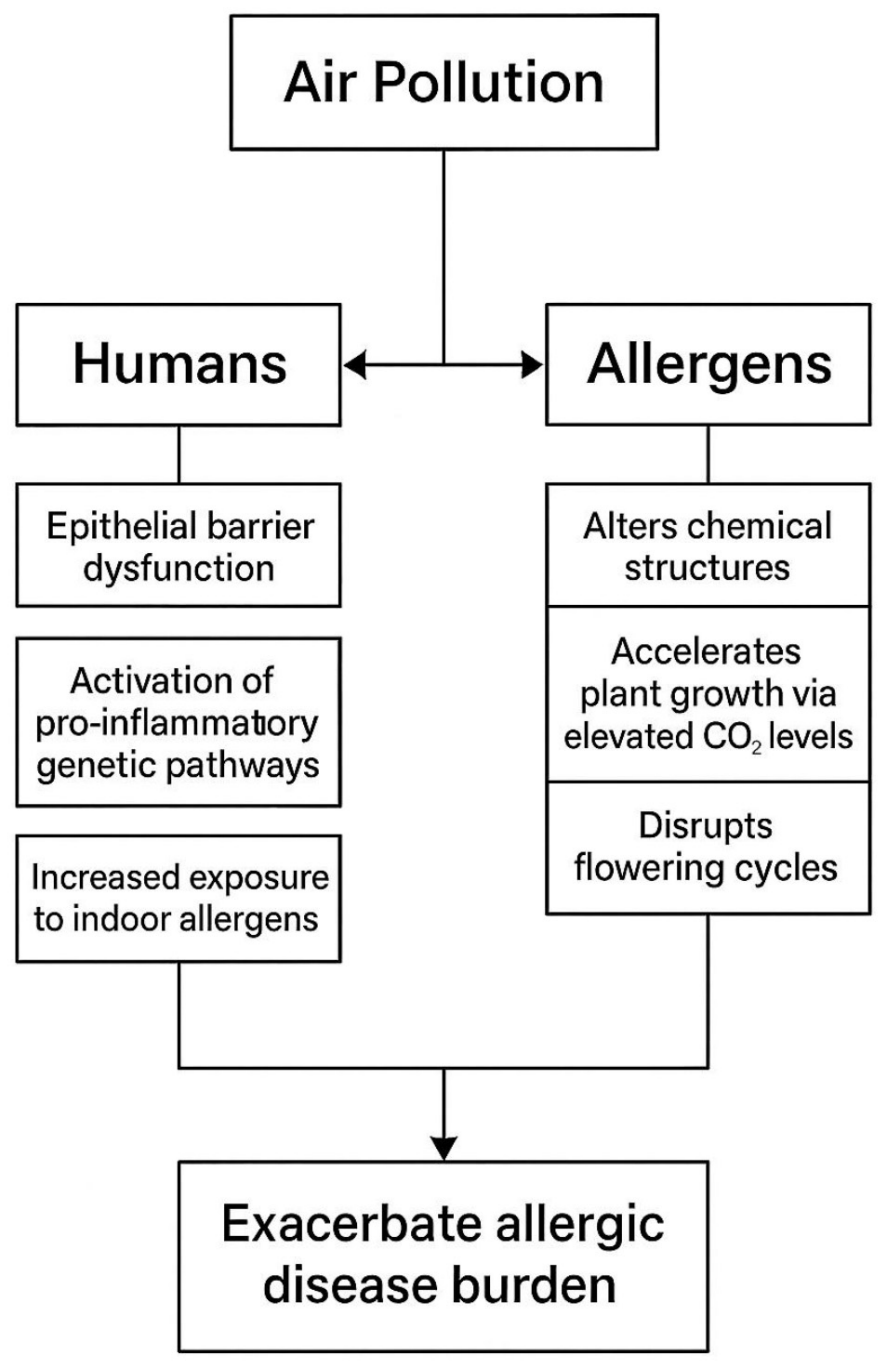
Multifactorial impact of air pollution on allergic disease burden.

Figure 1 shows multifactorial impact of air pollution on allergic disease burden.

Pollution can also increase susceptibility to respiratory infections through barrier disruption and altered immune responses (3, 10). Conversely, respiratory infections in early life (e.g., HRV, RSV) may predispose to asthma development, suggesting synergistic interactions among infections, allergens, and pollutants (10). Childhood exposure to environmental pollutants has been associated with increased risk of later immunological disorders (e.g., asthma, rhinitis, eczema, food allergy) (23). Food allergy prevalence has increased over recent decades, and changes in atmospheric CO_2_ and plant protein composition have been proposed as contributing factors, although individual exposure assessment remains challenging and causal attribution complex (22, 62, 66).

Finally, atmospheric pollution contributes to cancer risk. Particulate matter is recognized as a class I carcinogen, and pollutants may be metabolized by the skin microbiome into potentially carcinogenic compounds (58, 60, 63). Climate-related impacts on stratospheric ozone (historically linked to chlorofluorocarbons) can increase ultraviolet radiation exposure, a known carcinogenic factor. Estimates suggest that a 1% reduction in ozone thickness could increase melanoma risk by ~1%−2% and raise risks of squamous and basal cell carcinoma by several percent (48, 60, 67). UV exposure may also disrupt skin microbiota and promote inflammatory dermatoses via oxidative stress and sebaceous hyperplasia (58).

#### 3.2.4 Water and soil pollution

Persistent chemicals such as pesticides and pharmaceuticals can re-enter human exposure pathways through contaminated water and food. WHO emphasizes biomonitoring of such exposures and the importance of poison control capacity to protect populations (50). Beyond direct toxicity, many compounds act as endocrine disruptors and can alter endocrine–metabolic homeostasis (59). Environmental contamination with antibiotic compounds is also a key concern, as it may promote the development and dissemination of antimicrobial resistance (50).

#### 3.2.5 Extreme atmospheric events

##### 3.2.5.1 Storms, hurricanes, floods, and droughts

Climate change increases the frequency and alters the distribution of storms, floods, droughts, and hurricanes, producing immediate and long-term hydrogeological impacts (48, 53) (Table 4). Crop destruction and livestock losses can lead to food insecurity and malnutrition, with pediatric consequences ranging from micronutrient deficiencies to severe syndromes such as marasmus and kwashiorkor, particularly in areas undergoing desertification (60, 66, 68). Floods and droughts can also increase the risk of crop and water contamination, gastrointestinal infections, and toxic exposures, while creating standing water that supports vector breeding (48, 53, 66). Following floods or hurricanes, displacement into crowded shelters with limited hygiene can facilitate transmission of infectious diseases and skin infestations (e.g., scabies, pediculosis, mycoses), and sustained humidity can destabilize skin homeostasis and exacerbate dermatoses (27). Some reviews have not found a statistically significant association between floods and mortality, potentially due to limited inclusion of nutritional pathways and longer-term downstream effects (5).

**Table 4:** Primary health and environmental consequences associated with extreme weather events such as floods, droughts, and hurricanes.

**Extreme weather events**
**Health impacts**
• Destruction of crops and livestock, leading to malnutrition
• Contamination of crops and aquifers, resulting in gastrointestinal infections
• Formation of swampy areas where insect vectors concentrate
• In the case of floods, crowding in makeshift shelters favors the transmission of infectious diseases
**Environmental impacts**
• Destruction of structures and nature, with diffusion of carcinogenic substances
• Destruction of biodiversity

Biodiversity loss following extreme events may reduce microbial diversity in human-environment contact, with potential consequences for immune development—especially in childhood—when immune tolerance is being established. Lower biodiversity has been associated with higher prevalence of allergic and inflammatory diseases, whereas farm environments have been linked to lower rates of allergy in some contexts (62). Alterations in the gut microbiome have also been associated with systemic effects beyond the gastrointestinal tract, including correlations with neurological disorders (62).

A notable climate-related respiratory phenomenon is thunderstorm asthma, characterized by spikes in asthma exacerbations following thunderstorms during high-pollen seasons. Mechanistically, pollen rupture due to osmotic shock and storm-related forces can generate respirable allergen microparticles, triggering severe symptoms in sensitized individuals (49, 55, 57, 62, 65). Storms and hurricanes can also transport inhalants over long distances, shifting the seasonality and geography of allergic symptoms through new sensitizations and cross-reactivity (47, 48, 59, 60). Increased humidity during storms and floods may promote indoor mold growth and spore dissemination (49, 57).

Extreme events also affect mental health and endocrine–metabolic pathways. Stress responses may influence neurodevelopment and contribute to inflammatory states implicated in autoimmune risk (68). Psychological outcomes vary with event severity, coping capacity, and social context; populations at increased risk include children, older adults, economically disadvantaged individuals, first responders, and those with pre-existing mental health disorders or displacement (53, 69). Terms such as “solastalgia” (distress linked to environmental loss) and “eco-anxiety” (anxiety about climate change) reflect the growing relevance of climate-related psychological burden (69). These effects may require both acute and long-term monitoring and support, particularly for children, who also face increased trauma risk during disasters (57, 66, 69). Damage to infrastructure can increase environmental contamination, and disruption of healthcare delivery can worsen outcomes for chronic and oncologic patients (63).

##### 3.2.5.2 Fires

Rising temperatures increase wildfire risk. Fires emit carbon monoxide, nitrogen oxides, volatile organic compounds (e.g., formaldehyde, benzene), and large quantities of particulate matter (49). PM10 concentrations during fires may rise 1.2–10-fold, exacerbating asthma and chronic obstructive pulmonary disease, while PM2.5 can induce neutrophilic airway inflammation and systemic inflammatory responses linked to cardiopulmonary risk (49, 53, 57). Smoke impacts extend beyond local regions, as fine particulates can be transported long distances (reported up to 332 km), complicating exposure patterns (49, 66). Attributing health effects to specific fire-derived pollutants remains challenging because wildfire-related pollution can be difficult to distinguish from other sources (5). Pediatric vulnerability appears higher, and children with elevated BMI may face additional risk (56).

##### 3.2.5.3 Sandstorms

Higher temperatures contribute to sandstorms, including dust transport from the Sahara to parts of Europe, Asia, and the Americas. These events carry particulate matter, pollen, and microorganisms and have been associated with increased hospitalizations for asthma and COPD exacerbations, as well as cardiovascular and cerebrovascular events (49).

#### 3.2.6 Risk categories

**Figure 2:**
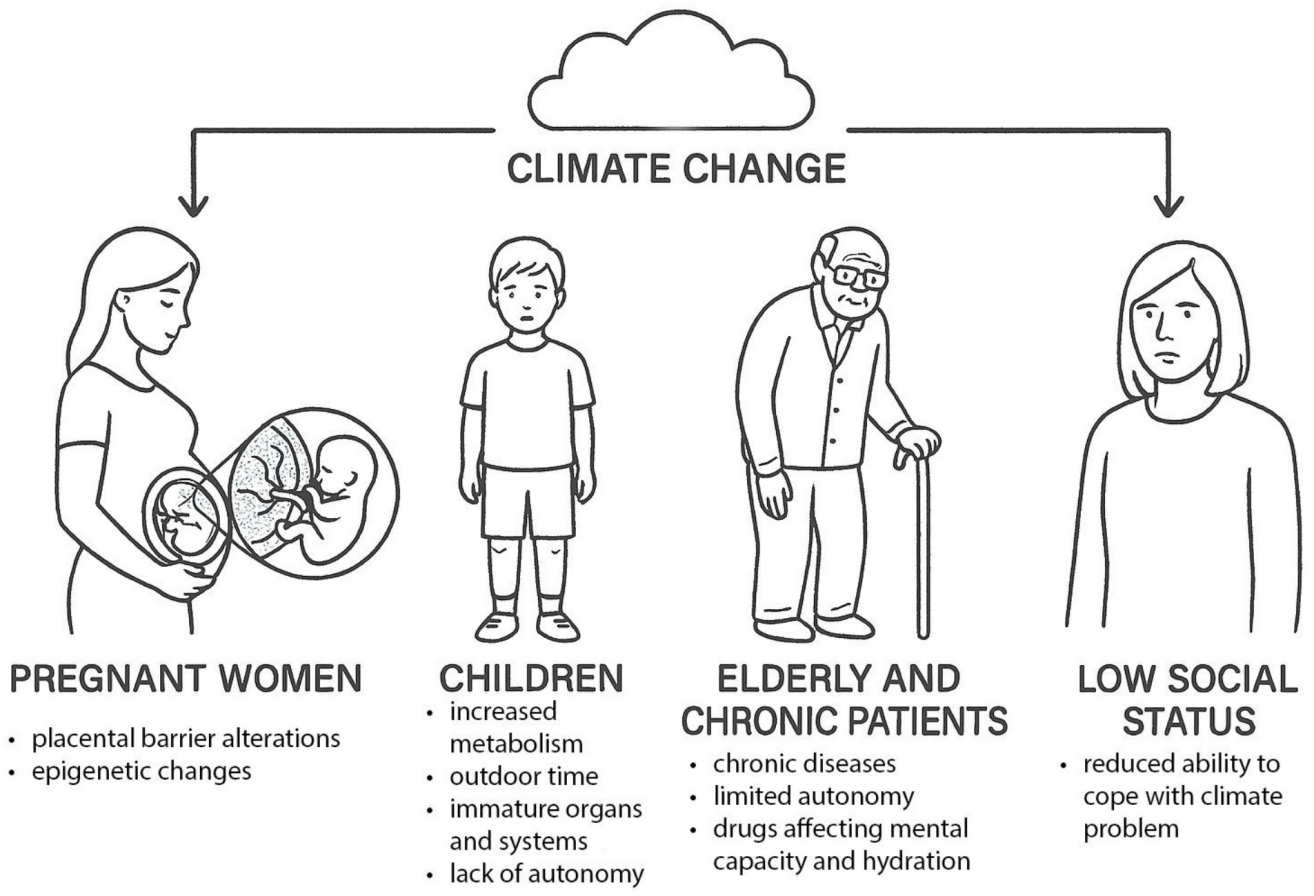
Groups most vulnerable to climate change.

Although climate-related risks affect all populations, susceptibility varies by age, physiology, social context, and baseline health. Infants and young children are among the most vulnerable (49, 70) (Figure 2). During pregnancy, exposure to pollutants can disrupt placental function and induce epigenetic changes with potential transgenerational consequences, contributing to adverse perinatal outcomes such as preterm birth and low birth weight (49, 70). Prenatal stress from extreme events may further affect fetal development (49, 70).

After birth, children's higher metabolic rates, greater intake of air, food, and water per kilogram of body weight, frequent outdoor activity, and hand-to-mouth behaviors increase exposure to pollutants and pathogens. Their immune and detoxification systems remain immature, heightening vulnerability to both direct toxic effects and infection (55, 57, 70). Exposure and impact also depend on developmental stage and event severity (49). Infants' higher needs for water and nutrition increase sensitivity to food-system disruptions, water scarcity, and contamination. Children's dependence on caregivers for protective behaviors during heatwaves, pollution peaks, and disasters further increases risk, especially for those requiring medical devices (49, 66).

Climate change has also been linked to pediatric mental health impacts, mediated by both biological effects of pollutants on neurodevelopment and psychosocial stress from disasters, displacement, and restrictions on daily activities. Catastrophic events may contribute to behavioral changes, depression, social withdrawal, and altered attachment patterns, and climate-related future anxiety may emerge around late childhood as awareness increases (26). In this context, pediatric healthcare professionals play a key role in counseling families, tailoring preventive guidance to the child's environment, and advocating for measures that reduce climate-related health risks (26). Climate change affects pediatric mental health through interconnected environmental, social, and health-system pathways, consistent with a One Health perspective. Acute climate-related events such as floods, heatwaves, and wildfires can precipitate anxiety, depression, post-traumatic stress symptoms, and behavioral changes in children, while chronic stressors including food and water insecurity, displacement, ecosystem degradation, and economic instability contribute to longer-term psychological distress (51). Concepts such as eco-anxiety and solastalgia reflect children's emotional responses to perceived environmental loss and anticipated climate threats, particularly among adolescents. These effects are amplified by children's developmental vulnerability, dependence on caregivers, and sensitivity to disruptions in social, educational, and healthcare systems (51).

A One Health–oriented response highlights the need to integrate pediatric mental health into climate adaptation, disaster preparedness, and community resilience strategies (53). Mental health and psychosocial support should be embedded within disaster response and recovery efforts, alongside medical care, shelter, and environmental remediation. School-based resilience and climate-literacy programs can strengthen coping skills, social connectedness, and adaptive capacity, while community education initiatives may support caregivers and reduce stigma surrounding mental health (53). Despite increasing recognition of these challenges, evidence on effective, scalable interventions for pediatric mental health in the context of climate change remains limited. Further interdisciplinary research is needed to evaluate integrated, child-centered adaptation strategies that address environmental stressors, social determinants, and health system responses simultaneously, ensuring that mental wellbeing is fully incorporated into One Health approaches to climate change.

### 3.3 Emerging infectious diseases

Climate change—persistent alterations in regional and global weather patterns over decades or longer—has driven profound environmental shifts that increasingly shape infectious disease emergence and re-emergence. It is now recognized as a major threat to human health, particularly through its effects on vector-borne diseases (VBDs), which are expanding in geographic range, seasonality, and transmission intensity (71). Globally, VBDs account for an estimated 219 million cases and more than 500,000 deaths annually, with a disproportionate burden among children, especially in Africa (72).

VBD transmission cycles depend on complex interactions between arthropod vectors, pathogens, vertebrate hosts, and environmental conditions. Some pathogens circulate predominantly in human-amplified cycles involving a limited number of competent vectors and humans as the principal reservoir, as occurs for many dengue virus (DENV) genotypes (64). In contrast, many VBDs are zoonotic, maintained in enzootic cycles involving wildlife reservoirs with occasional spillover to humans, as exemplified by West Nile virus (WNV), which primarily circulates among avian hosts (73). Europe, historically considered at relatively low risk for many arboviral infections, is increasingly reporting autochthonous transmission of pathogens such as dengue, chikungunya, Zika virus, and WNV, reflecting changing ecological suitability and growing vector establishment (74) (Table 5).

**Table 5:** Climate-sensitive vector-borne diseases relevant to Europe and pediatric populations.

**Disease**	**Pathogen**	**Main vectors**	**Reservoir hosts**	**Cycle type**	**Distribution**	**Pediatric impact**	**Climate/human drivers**	**Prevention/control**
Dengue (DENV-1–4)	*Flavivirus* (Flaviviridae)	*Aedes aegypti, A. albopictus*	Humans	Human–mosquito–human	Tropics; local cases in France, Italy	Often mild; risk of severe dengue (DHF/DSS)	↑ Temperature → faster EIP; ↑ urbanization, travel	Vector control; personal protection; vaccination (TAK-003, CYD-TDV)
Zika virus (ZIKV)	*Flavivirus* (Flaviviridae)	*A. aegypti, A. albopictus*	Primates, humans	Mosquito-borne; also sexual, vertical	Africa, Americas, Asia, Oceania; imported in Europe	Mild/asymptomatic; congenital Zika syndrome	↑ Temperature and urbanization → vector expansion northward	Vector control; travel precautions; pregnancy protection
Chikungunya virus (CHIKV)	*Alphavirus* (Togaviridae)	*A. aegypti, A. albopictus*	Non-human primates	Sylvatic & urban	Africa, Asia, Americas; local in Italy, France	Fever, rash, arthralgia; neonatal severe cases	E1-A226V mutation ↑ adaptation to *A. albopictus*; warmer climate ↑ range	Vector surveillance; traveler monitoring; source reduction
West Nile virus (WNV)	*Flavivirus* (Flaviviridae)	*Culex* spp.	Birds; humans/horses dead-end	Enzootic bird–mosquito	Endemic in S and C Europe; outbreaks in Italy, Greece, Hungary	Usually mild; rare neuroinvasive disease	Heatwaves, drought ↑*Culex* breeding; altered bird migration	Vector control; surveillance; equine vaccination

VBD dynamics are governed by multiple interacting determinants, including vector competence, host availability, human behavior, and health system capacity. Because vectors are ectothermic, their development, survival, biting rates, and the extrinsic incubation period (EIP) of pathogens are strongly influenced by environmental conditions, particularly temperature and humidity. Consequently, climate change has major implications for transmission patterns and outbreak risk (71). Global warming is of particular concern: the current ~1.1 °C rise approaches thresholds beyond which changes in Earth systems may become self-reinforcing and potentially irreversible (75). These changes are expected to increase climatic instability, with consequences such as intensified heatwaves, droughts, floods, and cyclones—events that can disrupt ecosystems, alter vector habitats, and reshape host–vector contact rates (76).

Beyond climate parameters alone, broader anthropogenic environmental transformations are tightly linked to emerging infections. Deforestation, agricultural expansion, intensive livestock production, land conversion, resource extraction, and urbanization can profoundly affect both the distribution of infections and the biology of vectors by modifying habitat suitability, reservoir composition, and transmission opportunities (71) (Table 6). Land-use change also contributes to climate change by promoting biodiversity loss and reducing carbon sequestration capacity. Importantly, alterations in land cover have been identified as key drivers of zoonotic spillover from wildlife to humans. Vector-borne diseases are particularly sensitive to these shifts because they influence host and vector density, predator abundance, availability of larval and adult habitats, local microclimates, and frequency of vector–host interactions (77). Deforestation in tropical regions may favor vectors that tolerate anthropogenic disturbance and can bridge transmission among wildlife, domestic animals, and humans; however, spillover typically requires multiple ecological, immunological, and behavioral conditions to coincide. Conversely, human–wildlife interactions may also facilitate reverse zoonosis (spillback), enabling pathogens to establish new enzootic cycles in wildlife populations (78).

**Table 6:** Framework linking climate and anthropogenic change to vector-borne disease emergence.

**Factor**	**Key drivers**	**Mechanisms**	**Epidemiological outcomes**	**Pediatric/public-health impact**
Climate factors	Temperature rise, altered rainfall, humidity changes, extreme events	Affect vector development, biting rate, survival, and pathogen extrinsic incubation period (EIP)	Extended transmission season; northward spread of tropical vectors	Earlier onset of arboviral season; higher exposure of outdoor-active children
Environmental and land-use change	Deforestation, agricultural expansion, irrigation, water storage, urbanization	Modify habitats, reservoir populations, and breeding sites	New ecological niches for *Aedes, Culex, Phlebotomus* spp.	Urban heat-island effect ↑*Aedes* density near schools/homes
Biodiversity and host dynamics	Reservoir displacement, reduced predator diversity, increased contact with wildlife	Facilitate spillover and spillback cycles	Emergence of zoonotic pathogens (WNV, leishmaniasis)	Increased child exposure during outdoor play/agricultural labor
Socioeconomic and demographic factors	Population mobility, informal housing, sanitation deficits	↑ Human–vector contact; poor access to prevention	Clustering of cases in peri-urban areas	Higher infection burden in marginalized children
Health-system factors	Surveillance, diagnostics, vaccination, vector-control programs	Influence detection and containment speed	Delayed response → outbreak amplification	Pediatric morbidity/mortality ↑ during delayed interventions

Urbanization is a major driver of contemporary VBD expansion. Rapid urban growth promotes proliferation of mosquito vectors such as *Aedes* spp. (*Ae. aegypti* and *Ae. albopictus*), *Culex* spp., and *Anopheles* spp., through increased human density, water storage practices, waste accumulation, and abundant artificial breeding sites. *Ae. aegypti*, in particular, is highly adapted to human-associated habitats and preferentially feeds on humans, supporting sustained urban transmission (71, 79). The ecological success of mosquitoes in cities is reinforced by plentiful larval habitats and reduced predation pressures (80). In addition, urban “heat islands” elevate local temperatures relative to surrounding rural areas, which may intensify heat-related morbidity and simultaneously enhance vector survival and pathogen development, thereby altering transmission dynamics (71, 81, 82).

In addition to arboviruses, other climate- and environment-sensitive infections of concern include malaria and leishmaniasis (parasitic diseases transmitted by insect vectors), tick-borne infections (e.g., Lyme disease, babesiosis, Crimean–Congo hemorrhagic fever, and rickettsioses), and waterborne diseases such as cholera caused by *Vibrio cholerae*. The recent global spread of mpox also highlights the evolving landscape of emerging infections. Collectively, climate-driven shifts in habitat suitability and species distributions, combined with increasing anthropogenic pressures on ecosystems, can disrupt biodiversity and facilitate new opportunities for pathogen emergence and transmission (83, 84). Despite growing concern, comprehensive analyses focusing specifically on VBD emergence in Europe and pediatric impacts remain limited.

#### 3.3.1 Flaviviridae

Among emerging and climate-sensitive pathogens, viruses belonging to the *Flaviviridae* family represent a major and growing threat to child health due to their expanding geographic distribution and capacity to cause both acute and long-term complications.

Zika virus (ZIKV) is a mosquito-borne infection caused by a positive-sense, single-stranded RNA virus in the genus *Flavivirus* (family *Flaviviridae*), within the *Spondweni* serocomplex. ZIKV is primarily transmitted by *Ae. aegypti* and *Ae. albopictus*, but sexual transmission, vertical transmission (mother-to-fetus), and transfusion-associated transmission have also been documented (85).

Prior to 2007, only 14 human cases had been reported, largely in Africa and Asia. Since 2013, major outbreaks occurred in the Pacific (including French Polynesia) and subsequently in Brazil, where the 2015 epidemic was estimated to involve ~1.3 million infections, followed by widespread dissemination across the Americas (86–90). In Italy, 12 imported cases were reported in 2016 (91), and four imported cases were reported in 2025 (92). After the 2015–2017 epidemic period, no major outbreaks have been documented, although prediction remains difficult due to under-reporting of asymptomatic infection, cross-protection among flaviviruses, and uncertainty regarding the duration of post-infection immunity (93). ZIKV currently circulates in Africa, the Americas, Asia, and Oceania, with imported cases reported across Europe (86, 94–98).

Because *Aedes* mosquitoes tolerate higher temperatures and thrive in urban environments, warming and urbanization may facilitate further range expansion. Projections suggest that by 2080, more than one billion additional people—mainly in North America and Europe—could be exposed to *Aedes*-borne arboviruses, including ZIKV (99). Several *Aedes* species beyond *Ae. aegypti*, including *Ae. albopictus* and others, have demonstrated vector competence under field or experimental conditions (91, 92). Non-vector routes include vertical, intrapartum, sexual, transfusion, and transplant-associated transmission (91–93).

After an infectious bite, the EIP is typically 3–12 days. Most infections are mild; in children, symptoms include fever, asthenia, headache, rash, conjunctivitis, and arthralgia (100). Severe neurological complications (e.g., Guillain–Barré syndrome, meningoencephalitis, myelitis, optic neuritis) are uncommon, but the most serious consequences arise during pregnancy, where fetal infection can cause congenital Zika syndrome, including microcephaly and other neurodevelopmental and ocular abnormalities (101–103). Diagnosis relies primarily on RT-PCR; viremia is usually detectable in serum and saliva for 3–7 days after symptom onset and in urine up to 2–3 weeks. Prevention focuses on personal protective measures, travel precautions, and vector control through breeding-site reduction (104–107).

WNV is a mosquito-borne zoonotic arbovirus within the genus *Flavivirus* (family *Flaviviridae*). First isolated in 1937 in Uganda, it is now the most widely distributed flavivirus globally, present in Africa, Asia, Europe, Australia, and the Americas (108). WNV is maintained in an enzootic cycle between birds (amplifying hosts) and mosquitoes, mainly *Culex* spp.; humans and horses are incidental “dead-end” hosts due to insufficient viremia to sustain onward mosquito infection (109, 110).

WNV includes at least nine lineages, with lineages 1 and 2 most commonly associated with human disease. Lineage 1A circulates across Europe, the Middle East, Africa, West Asia, and North America, while lineage 2—formerly confined to sub-Saharan Africa—has expanded into Europe (111). Transmission is seasonal, typically peaking in late summer, and is promoted by drought, stagnant water, and high temperatures that enhance mosquito abundance and viral amplification (112, 113).

European outbreaks have been repeatedly linked to climatic anomalies. Italy reported equine cases in 1998 and human cases beginning in 2008, with subsequent outbreaks associated with heatwaves (114, 115). The 2018 season showed unprecedented case numbers, attributed to sustained heat and ecological disruption (116, 117). Warming may shorten EIP, extend mosquito breeding seasons, and alter bird migration patterns, contributing to northward expansion, including detection of WNV in the Netherlands in 2020 (82, 115, 118).

Approximately 80% of infections are asymptomatic; around 20% develop West Nile fever. Less than 1% progress to neuroinvasive disease (meningitis, encephalitis, acute flaccid paralysis), more commonly in older or immunocompromised individuals (119). Pediatric infections are usually mild, but severe cases with long-term sequelae have been described (120, 121). No specific antiviral therapy exists; management is supportive (122). Prevention relies on vector control and personal protection. Although equine vaccines are available, no licensed human vaccine exists, though candidates are under development. Climate-sensitive surveillance remains a key public health strategy (111, 123).

Dengue is caused by four related serotypes (DENV-1–4) within the genus *Flavivirus* and is transmitted mainly by *Ae. aegypti* and *Ae. albopictus* (124, 125). Infection confers lifelong immunity to the infecting serotype but only transient cross-protection; secondary infection with a different serotype can increase risk of severe disease via antibody-dependent enhancement (126). Globally, dengue burden is highest in the Asia–Pacific region (~70%), followed by Africa and the Americas (127). Although not endemic in Europe, the presence of competent Aedes vectors has enabled local transmission alongside imported cases. Italy reported 185 cases in 2019 and 450 cases in 2024 (425 imported, 25 autochthonous), with no deaths (127–129).

Dengue incidence has increased sharply due to urbanization, globalization, sanitation gaps, and mobility. WHO estimates ~390 million infections annually across 128 countries, and projections suggest 5–6 billion people could be at risk by 2050 (130, 131). Climate change influences dengue by accelerating vector development, shortening EIP, and increasing transmission efficiency (125, 132). Reduced mobility during the COVID-19 pandemic corresponded with lower dengue incidence, underscoring the role of behavioral and mobility patterns in transmission (133). Risk is shaped by rainy-season vector abundance, shortened EIP at higher temperatures, dense susceptible populations, and prolonged human viremia; some models estimate that each 1 °C rise may increase infection risk by ~13% (131, 134–137).

Epidemic dengue often follows sporadic introductions, whereas hyperendemic settings with multiple serotypes tend to shift symptomatic disease toward pediatric populations due to higher adult immunity (138–141). *Ae. albopictus* contributes to transmission in temperate regions but is generally less anthropophilic and less efficient than *Ae. aegypti* (126, 134, 142–144). Co-circulation with Zika and chikungunya may also occur because the vectors overlap (145).

Clinically, most infections are asymptomatic. Symptomatic dengue typically presents after 4–10 days with fever, rash, myalgia, arthralgia, retro-orbital pain, and gastrointestinal symptoms; severe dengue (DHF/DSS) can involve plasma leakage, hemorrhage, and shock, with mortality < 1% with appropriate care (146, 147). Pediatric presentations may be less specific; irritability may replace typical pain syndromes, and studies report higher rates of rash, diarrhea, hepatomegaly, and convulsions compared with adults (55, 148–150). Treatment is supportive, emphasizing careful fluid management and antipyresis (151). Two vaccines are licensed: CYD-TDV is recommended only in seropositive individuals due to increased severe dengue risk in seronegative recipients, while TAK-003 (Qdenga) has been approved in multiple settings, including the EU for individuals ≥4 years (152–156).

#### 3.3.2 Togaviridae

*Togaviridae* comprise a family of positive-sense, single-stranded RNA viruses that include several medically important arthropod-borne pathogens affecting humans. CHIKV is an RNA virus in the genus *Alphavirus* (family *Togaviridae*), transmitted mainly by *Ae. aegypti* and *Ae. albopictus*. Non-human primates act as reservoirs, while humans serve as amplifying hosts during epidemics (109, 110). CHIKV is endemic in tropical and subtropical regions, and includes major lineages such as West African and East/Central/Southern African (ECSA), the latter giving rise to the Asian genotype (119, 157, 158). Since its discovery in 1952, CHIKV has caused numerous outbreaks; the first documented autochthonous European outbreak occurred in Ravenna, Italy (2007) following importation by a viremic traveler (159). Autochthonous transmission was also documented in France in 2010, including pediatric cases, implicating *Ae. albopictus* (160, 161). Climate suitability for *Ae. albopictus* in Europe is expected to expand under warming scenarios (162, 163), and additional outbreaks were reported in Italy in 2017 (164).

Viral adaptation, notably the E1-A226V mutation, has enhanced replication in *Ae. albopictus*, facilitating transmission in temperate climates and supporting the expansion of CHIKV risk in Europe (165–167). The EIP is temperature-dependent; while robust data exist for dengue, comparable experimental EIP data for CHIKV remain limited, and few studies have modeled European spatiotemporal dynamics under climate scenarios (161).

Clinically, CHIKV typically causes fever, rash, headache, myalgia, and severe arthralgia; persistent joint pain may last weeks to months. Severe complications are uncommon but may involve neurological or cardiovascular disease, especially in neonates or individuals with comorbidities (119, 159, 167). Neonates born to viremic mothers are at risk of severe illness and neurological sequelae (168). Diagnosis relies on RT-PCR during the acute phase or serology thereafter; management is supportive, and prevention depends on vector control and personal protection (169). Pediatric surveillance is important because children may face increased risk as vector distributions shift and outbreaks occur in new regions (170, 171).

#### 3.3.3 Vector-borne diseases and pediatric impact

Children are among the most vulnerable populations to climate-related infectious threats, including vector-borne and zoonotic diseases. Their developing immune systems, higher metabolic rates, thinner skin, and behavioral patterns (e.g., outdoor play) increase exposure and may heighten risk of severe outcomes (88–90). Climate change influences vector distribution, abundance, and seasonality: warmer temperatures and increased humidity can accelerate mosquito breeding, shorten incubation periods, and extend transmission seasons. In Europe, these processes have supported the establishment and spread of *Ae. albopictus* and, in some settings, *Ae. aegypti*, increasing the potential for local outbreaks of dengue, chikungunya, and Zika. Similarly, northward expansion of *Phlebotomus* spp. raises concerns about leishmaniasis emergence in previously unaffected regions.

The pediatric burden is multifaceted. Beyond acute infection, VBDs can contribute to malnutrition, school absenteeism, long-term disability, and financial hardship for families. In many settings, prevention strategies (vector control, vaccination policies, surveillance) are not specifically tailored to pediatric needs, contributing to delays in diagnosis and treatment. Effective mitigation therefore requires child-centered surveillance, preventive education, timely clinical recognition, and integrated One Health preparedness. Predictive models incorporating climate, entomological, and demographic data may help identify emerging risk zones and guide targeted public health responses (87, 91, 93).

## 4 Discussion

Despite increasing recognition of the interconnections between AMR, child health, and the One Health paradigm, major knowledge and implementation gaps persist. Few studies have explicitly centered children within One Health AMR frameworks, and longitudinal cohort designs linking antibiotic exposure in children with animal contact, environmental reservoirs, and downstream resistance outcomes remain rare (17, 20). Similarly, the transmission dynamics of specific resistant pathogens across the human–animal–environment interface require deeper investigation. In particular, wider application of molecular epidemiology and strain-typing approaches (e.g., whole-genome sequencing, plasmid tracking) would help clarify whether resistant organisms and resistance genes detected in pediatric infections overlap with genotypes circulating in livestock, companion animals, water, and soil (22, 23). Pediatric outcomes also remain insufficiently characterized: data on treatment failure, morbidity, mortality, and long-term sequelae attributable to resistant infections are limited, especially in LMICs, where diagnostic gaps and under-reporting are common (9, 26, 43). Beyond epidemiology, economic and implementation research is urgently needed to determine which One Health interventions are feasible, scalable, and cost-effective in resource-constrained settings, and how they can be integrated into existing child health and public health programmes (17, 45). Finally, alternatives to antibiotics—including vaccines, probiotics, and phage-based approaches—are promising across human, animal, and environmental domains but remain at an early stage of translation and evaluation in pediatric settings (10). Systems-thinking and participatory models that map AMR drivers and leverage points may be particularly valuable for identifying child-specific exposure pathways and prioritizing actionable interventions across sectors (46).

Taken together, these findings reinforce the importance of framing pediatric AMR within a One Health paradigm. Children's risk is shaped not only by antibiotic prescribing and healthcare exposures, but also by household and community contexts, including interactions with animals, food systems, water and sanitation, and environmental contamination (13, 22, 23). A child-centered One Health strategy should therefore combine age-disaggregated surveillance, pediatric-adapted antimicrobial stewardship, strengthened infection prevention and control, action to reduce non-therapeutic antibiotic use in animals, environmental monitoring and remediation, caregiver and community education, and coordinated cross-sector governance (22, 24, 45). Without embedding children into national and global AMR agendas, a highly vulnerable population will remain exposed to the dual pressures of resistant infections and ecosystem-driven transmission.

Beyond AMR, climate change, pollution of air, water, and soil, and biodiversity loss represent intertwined threats with direct and indirect consequences for health. Predicting the net impact of rising temperatures on morbidity and mortality remains challenging, partly because outcomes depend on adaptive capacity, infrastructure, and access to protective technologies (52). Comparisons across studies are also limited by heterogeneity in exposure metrics and the lack of universally defined temperature thresholds. Nonetheless, evidence suggests that morbidity and mortality increase with the intensity and duration of extreme heat events, supporting the value of establishing regionally appropriate temperature thresholds and alert systems for population protection (53).

Public health responses to climate-related risks are typically framed as mitigation (reducing the drivers of climate change) and adaptation (reducing exposure and vulnerability). Mitigation strategies include transitioning to renewable energy, improving energy efficiency, and promoting sustainable transport and food systems. Adaptation strategies include heat-health action plans, disaster preparedness, improved ventilation and building design, surveillance and early warning systems, vector control measures (e.g., source reduction, mosquito nets), safe water and sanitation interventions, and health sector actions to reduce environmental footprints (50, 56, 57, 61). Ensuring equitable access to potable water, sanitation services, safe waste and wastewater disposal, and resilient transportation and healthcare infrastructure is foundational, particularly for children and other high-risk groups (4). Expanding urban green spaces may also support child health by reducing heat exposure and promoting healthier microbial and environmental ecosystems, although implementation must be context-specific and aligned with broader planning policies (62).

At the policy level, WHO has emphasized the health burden of environmental risk factors and the importance of coordinated action linking climate change, pollution, and biodiversity loss within a One Health perspective. The seventh WHO ministerial conference on environment and health (Budapest, July 2023) reinforced these priorities and positioned the “Roadmap for healthier people, a thriving planet, and a sustainable future 2023–2030” as a guiding framework for member states to reduce health impacts of environmental change (4). Health professionals—particularly pediatricians—play a crucial role in translating these priorities into practice by educating families, supporting preventive behaviors, and advocating for policies that reduce exposure and improve resilience among children (55). Educational initiatives are also needed: structured training on climate change and child health in medical curricula and pediatric residency programs has been recommended to prepare clinicians to recognize, manage, and prevent climate-sensitive health risks across diverse contexts (172).

Strengthening One Health approaches is equally essential for mitigating climate-sensitive infectious diseases. Surveillance systems should integrate entomological, clinical, laboratory, and climatic data to support early detection, rapid risk assessment, and timely response (71, 82). Education campaigns, targeted vector control, vaccination where available, and adaptation planning for changing vector ecology are key components of long-term prevention strategies (74, 123, 156). In Europe, rising temperatures, altered rainfall patterns, land-use change, and globalization of travel and trade are reshaping the distribution of arthropod vectors and enabling autochthonous transmission of pathogens previously considered tropical, reinforcing the need for sustained preparedness and cross-border coordination (74, 82, 115).

Priority actions should include reducing inequities in healthcare access and strengthening surveillance capacity in LMICs, where the greatest burden of climate-sensitive infections and AMR-related mortality is often concentrated (9, 26, 72). Surveillance expansion must be supported by affordable diagnostic and laboratory tools—including molecular and serological testing, and where feasible, genomic approaches—to improve detection and characterize transmission dynamics (43, 45). Digital innovations can further support real-time monitoring of importation risk, climate suitability, and spatial mapping of transmission hotspots to guide targeted interventions. Finally, sustained investment in interdisciplinary research is required to clarify the mechanisms linking climate variability, ecosystem change, and infectious disease dynamics, enabling evidence-based interventions that are effective, scalable, and equitable (75–77). Coordinated global governance—and fair implementation across countries and communities—will be indispensable to mitigate the accelerating emergence and spread of infectious diseases under climate change (45, 76).

This narrative review has several limitations that should be acknowledged. As a non-systematic review, it does not follow a formal protocol for study selection or quality assessment, and relevant evidence may have been inadvertently omitted. The available literature is also heterogeneous, with notable gaps in child-specific data, particularly from low- and middle-income countries, and limited evaluation of long-term outcomes and intervention effectiveness. In addition, evidence on integrated One Health interventions remains uneven across regions and thematic areas, constraining direct comparison and generalizability. Nevertheless, the strengths of this review include the breadth of topics covered, the integration of human, animal, and environmental health perspectives, and the inclusion of recent and emerging evidence. These features make the manuscript relevant to a wide multidisciplinary audience spanning pediatrics, infectious diseases, public health, environmental health, and policy.

## 5 Conclusions

Climate change is increasingly acting as a powerful catalyst for the emergence and re-emergence of vector-borne and zoonotic diseases in Europe, reshaping the geographic distribution, seasonality, and intensity of infectious threats that were once considered confined to tropical regions (71, 74, 82). Rising temperatures, altered precipitation patterns, land-use change, biodiversity loss, and urbanization collectively create ecological conditions that favor the establishment of competent vectors and the circulation of pathogens, with measurable consequences for human health. Within this evolving landscape, children represent a uniquely vulnerable population, due to their physiological susceptibility, developmental stage, behavioral exposures, and dependence on adults and systems for protection and care (2, 70).

This review highlights that the health risks faced by children in the context of climate change extend beyond infection alone. VBDs intersect with AMR, environmental pollution, food and water insecurity, and mental health stressors, reinforcing the need for integrated, child-centered responses. The One Health framework provides a unifying approach to understand and address these complex interactions by explicitly linking human, animal, and environmental health domains. However, current surveillance systems, research agendas, and policy frameworks insufficiently incorporate pediatric perspectives, leading to under-recognition of children's exposures, outcomes, and long-term consequences (9, 43).

Protecting child health in a changing climate therefore requires multidisciplinary and cross-sectoral collaboration among clinicians, pediatricians, epidemiologists, veterinarians, entomologists, ecologists, environmental scientists, and policymakers. Strengthened surveillance that integrates clinical, entomological, and climatic data; early-warning systems for climate-sensitive infections; targeted vector control; vaccination strategies where available; and pediatric-adapted antimicrobial stewardship are all essential components of an effective response (45, 71, 123). Equally important are investments in health system resilience, environmental protection, urban planning, sanitation, and access to clean water, particularly in socioeconomically disadvantaged settings.

Finally, children must be positioned not only as beneficiaries of protection but also as long-term stakeholders in planetary health. Education, community engagement, and empowerment of families and young people can foster awareness, resilience, and sustainable behaviors that extend benefits across generations. Advancing a child-centered One Health agenda is therefore not optional but imperative: without it, climate change will continue to amplify infectious disease risks and health inequities, undermining progress in child health and threatening the wellbeing of future generations (4, 50).
